# Mechanism for attenuated outward conductance induced by mutations in the cytoplasmic pore of Kir2.1 channels

**DOI:** 10.1038/srep18404

**Published:** 2015-12-18

**Authors:** Hsueh-Kai Chang, Masayuki Iwamoto, Shigetoshi Oiki, Ru-Chi Shieh

**Affiliations:** 1 Institute of Biomedical Sciences, Academia Sinica, Taipei 11529, Taiwan, ROC; 2 Department of Molecular Physiology and Biophysics, University of Fukui Faculty of Medical Sciences, Eiheiji-cho, Yoshida-gun, Fukui 910-1193, Japan

## Abstract

Outward currents through Kir2.1 channels regulate the electrical properties of excitable cells. These currents are subject to voltage-dependent attenuation by the binding of polyamines to high- and low-affinity sites, which leads to inward rectification, thereby controlling cell excitability. To examine the effects of positive charges at the low-affinity site in the cytoplasmic pore on inward rectification, we studied a mutant Kir channel (E224K/H226E) and measured single-channel currents and streaming potentials (V_stream_), the latter provide the ratio of water to ions queued in a single-file permeation process in the selectivity filter. The water-ion coupling ratio was near one at a high K^+^ concentration ([K^+^]) for the wild-type channel and increased substantially as [K^+^] decreased. On the other hand, fewer ions occupied the selectivity filter in the mutant at all [K^+^]. A model for the Kir channel involving a K^+^ binding site in the wide pore was introduced. Model analyses revealed that the rate constants associated with the binding and release to and from the wide-pore K^+^ binding site was modified in the mutant. These effects lead to the reduced contribution of a conventional two-ion permeation mode to total conductance, especially at positive potentials, thereby inward rectification.

Inward rectifier K^+^ channel (Kir2.x) subfamily members mediate inwardly rectifying K^+^ currents, which are important in the maintenance of stable resting membrane potentials, in controlling excitability and in shaping the final repolarization of action potentials in excitable cells^1^,^2^,^3^,^4^. Among the subfamily members, the Kir2.1 isoform determines the properties of cardiac inward rectifying currents when heteromeric complexes are formed^5^. The current-voltage relationship of Kir2.1 channels displays a unique hump-shape that can be attributed to the presence of an inward rectification mechanism and allows inward currents to pass through the channels more easily than outward currents.

The pore of a Kir2.1 channel is long and consists of a cytoplasmic pore, central cavity and selectivity filter (Fig. 1), which all have different widths. The mechanism underlying the inward rectification of Kir2.1 channels has been ascribed to the voltage-dependent block of outward currents by internal Mg^2+^ and polyamines^6^,^7^,^8^,^9^,^10^,^11^. Although outward Kir2.1 currents are much smaller than inward ones under physiological conditions, they control the excitability and repolarization duration in excitable cells, such as neurons and cardiac myocytes. Therefore, the process of inward rectification plays a critical role in the physiological functions of Kir2.1 channels.

Kir2.1 channels^12^ are blocked by polyamines with either high affinity in the central cavity or low affinity in the cytoplasmic pore^13^,^14^. The mechanisms for the high-affinity block has been clearly attributed to voltage-dependent block of central cavity by polyamines and Mg^2+^ 8,9,10,15,16, whereas the mechanism associated with the low-affinity block remains elusive. It has been shown that the inward rectification induced by polyamines interacting with E224 and E299 accounts for the low-affinity block^13^,^14^. Various mechanisms have been proposed to explain the low-affinity block in Kir2.1 channels. Some studies suggest that polyamines that are bound at the low-affinity site decrease K^+^ efflux via an electrostatic effect^17^,^18^,^19^,^20^. It has been proposed that E224 and E299 facilitate the entry and exit of polyamines to and from the final pore-plugging site located deeper in the pore and that internal blockers bind to E224 and E299 without occluding the pore^14^,^21^. The effect of electrostatic changes in the intracellular pore on channel conductance has been studied in several types of K^+^ channels^18^,^22^,^23^,^24^. The results of these studies suggest that local [K^+^] is lowered via an altered electrostatic potential at the cytoplasmic pore (Fig. 1), but these lines of evidence are indirect. Therefore, we aimed to examine the role of the selectivity filter in ion permeation when the cytoplasmic pore is modified.

The crystal structures of K^+^ channels have elucidated permeation mechanisms on an atomistic scale. In the selectivity filter, multiple ions and water molecules occupy defined sites^25^,^26^,^27^. Based on the equilibrium ion distribution in the crystal structure, Morais-Cabral *et al.* proposed an alternating placement of K^+^ ions (i) and water molecules (w) in the four binding sites (either i-w-i-w or w-i-w-i) of the selectivity filter^28^,^29^. These distribution patterns suggest that the ratio between water and ion fluxes (water-ion coupling ratio; *CR*_w-i_) is quantifiable, and we previously measured a coupling ratio of 1:1 at high [K^+^] for HERG and KcsA K^+^ channels^29^,^30^,^31^.

In the present study, we evaluated *CR*_w-i_ in the Kir2.1 channel by measuring *V*_stream_ and investigated the effects of introducing charges to the cytoplasmic entry on water-K^+^ coupling using a mutant channel. The *CR*_w-i_ value for the wild-type channel was around one at a high [K^+^], which is similar to other types of K^+^ channels^30^,^31^. Significant increases in the water-K^+^ coupling ratio were obtained for the mutant channel. To investigate the mechanisms underlying both the altered *CR*_w-i_ values in the mutant channel and the inward rectification, we created a permeation model that involves the K^+^-binding site in the cytoplasmic pore. The optimized models for the mutant and wild-type channels indicate how ions and water molecules are queued in the narrow and short selectivity filter during permeation and reveal the effect of electrostatic changes at the low-affinity site on outward K^+^ conductance in the Kir2.1 channel.

## Results

### Effects of mutations in the low-affinity site on single-channel conductance

To investigate the effect of introducing a positive charge at site 224 on K^+^ permeation, we compared the wild-type Kir2.1 channels with an E224K/H226E mutant channel. Previously, we showed that the macroscopic current-voltage curve of the E224K/H226E channel demonstrates strong inward rectification in the absence of intracellular blockers^18^. To further investigate whether the mutation affected ion permeation or gating, we compared single-channel currents of the wild-type and E224K/H226E channels in the absence of the intracellular blocker (Fig. 2a). At all of the voltages tested, the single-channel current was smaller in the mutant than in the wild-type channel. However, the probability of the opening of the E224K/H226E channel did not appear to be different from that of the wild-type channel. Single-channel currents were recorded at varying symmetrical [K^+^], and the current−voltage curve displayed strong inward rectification in the E224K/H226E mutant at all tested [K^+^] (Fig. 2b). The dependence of the single-channel conductance on symmetrical [K^+^] at −100 mV for the wild-type and mutant channels were fitted to the Michaelis-Menten equation, and the apparent binding affinities for K^+^ (K_M_) were 10 mM and 17 mM for the wild-type and mutant channels, respectively (Fig. 2c, broken lines). These results demonstrate that the inward rectification of the E224K/H226E channel occurs via changes in ion permeation rather than channel gating.

### Measurements of V_stream_

To examine the altered permeation mechanism in the mutant channel, we investigated water-K^+^ coupling ratios by measuring V_stream_. Figure 3a shows the experimental scheme in the inside-out patch with a pipette solution containing sorbitol (0.5–1.5 M). The osmolarity of the solution bathing both sides of the membrane was equilibrated beforehand [isotonic condition, where ΔOsm = Osm_out_ − Osm_in_ = 0; both pipette and internal solutions contained sorbitol (High-Osm), denoted as High-Osm_in_/High-Osm_out_]. When the intracellular solution was changed to one that contained no sorbitol (Normal-Osm), an osmotic gradient was established across the membrane (i.e., the hypertonic condition, Normal-Osm_in_/High-Osm_out_), which drove water outward. This water efflux generated a K^+^ efflux, even in the absence of electrochemical potential gradients for K^+^. This coupled water-K^+^ efflux yielded a negative shift of the reversal potential (V_rev_), which is referred to as V_stream_, after corrections of liquid junction potentials (Table 1; see Methods).

To measure V_stream_, a train of ramps was applied to an inside-out patch containing wild-type Kir2.1 channels (Fig. 3b). At the first and last six ramps in the train, the patch membrane was exposed to isotonic conditions (High-Osm_in_/High-Osm_out_), whereas at the six middle ramps, the patch was exposed to a hypertonic condition (Normal-Osm_in_/High-Osm_out_), which led to a net outward water flux. Kir2.1 currents increased during the hypertonic condition, and the effect was reversible upon return to the isotonic condition (Fig. 3b). The immediate increase in currents during the osmotic pulse confirmed the quick change in osmolarity. This increase of current in the hypertonic condition is likely due to a decrease in resistance in the cytoplasmic long pore after removal of sorbitol^32^. To better view the data, a ramp-set consisting of a positive and a negative sweep and current traces were enlarged (Fig. 3c). The first six traces (black line) and last six traces (cyan line) were superimposed, and the six middle traces (red line) overlapped with one another. The current-voltage curves that were obtained from the negative ramps were nearly identical to those from the positive sweeps, and the two branches crossed at the same V_rev_ (Fig. 3d). Because water flowed out with K^+^ through the channel pore when a hypertonic gradient was established across the membrane, V_rev_ shifted in the negative direction.

The time course of V_rev_ obtained from both the positive and negative ramp branches before, during and after the osmotic pulse are shown in Fig. 3e. V_rev_ shifted toward a negative value during water efflux (the hypertonic condition). After returning to the hyperosmotic condition, V_rev_ immediately and fully recovered to the original level (data points 13–18). This finding indicates that local K^+^ accumulations are not significant, likely because of the low water permeability of oocyte membrane. V_stream_ was determined as the difference in V_rev_ values immediately before and at the beginning of the osmotic pulse.

The same protocol was used to determine V_stream_ in the E224K/H226E mutant (Fig. 4a,b). Similar to the finding in the wild-type channel, V_rev_ shifted in the negative direction (Fig. 4c). However, the V_stream_ value was larger in the mutant (Fig. 4d) than in the wild-type.

Next, we examined in which part of the pore the water-K^+^ coupling occurred when an osmotic gradient was applied across the membrane. To address this question, it is essential to know the geometrical profile of the pore. The cytoplasmic pore and the central cavity are wide, which allow channel blockers, such as tetrabutylammonium, to access the central cavity of Kir2.1 channel^33^. It has been demonstrated that 2-(trimethylammonium) ethyl methanethiosulfonate (MTSET) accesses the central cavity of the wild-type Kir2.1^34^,^35^,^36^,^37^ and the E224K/H226E mutant^18^. Therefore, sorbitol molecules, which are smaller than MTSET, may advance through the cytoplasmic pore and access the central cavity in term of size consideration. In addition, the coupling ratio of the wild-type Kir2.1 is similar to that of a KcsA channel^30^, which, unlike the Kir2.1, lacks the long cytoplasmic pore. Therefore, it is likely that sorbitol is also accessible to the central cavity of the Kir2.1 channel in a way similar in the KcsA channel. Furthermore, even if sorbitol could not access to the central cavity, the results would not change substantially because the coupling of ions and water molecules is weak in the wide-pore region, and water flux through the pore makes a negligible contribution to the *CR*_w-i_ value^30^,^38^. Therefore, it is likely that the streaming file of water and K^+^ through the narrow selectivity filter generates V_stream_.

### Reduced K^+^ occupancy in the selectivity filter of the E224K/H226E mutant

To quantitate *CR*_w-i_ values from V_stream_, the relationships between V_stream_ and ΔOsm were plotted (Fig. 5a–c). The slope of the relationship increased as [K^+^] decreased in both the wild-type and mutant. Further, the slope was steeper in the mutant than in the wild-type at the same [K^+^]. *CR*_w-i_ values were calculated using the following equation^30^,^31^,^39^,^40^:


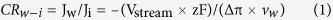


where Δπ is the osmotic pressure, *v*_*w*_ is the molar volume of water, *z* is the ion valence, and *F* is the Faraday constant.

We found that *CR*_w-i_ values increased when [K^+^] decreased (Fig. 5d). At lower [K^+^], water flow carried fewer K^+^ because the number of K^+^ ions occupying the selectivity filter was smaller than that of water molecules. As [K^+^] increased, *CR*_w-i_ values decreased and approached one, which indicated that approximately the same number of K^+^ ions and water molecules occupied the selectivity filter. This value supports the alternating occupancy of K^+^ and water (w-i-w-i or i-w-i-w) proposed by Morais-Cabral *et al.*^25^. Furthermore, these *CR*_w-i_ values are consistent with data previously reported for other types of K^+^ channels^30^,^31^, in which the permeation mode (described in the next section) is substantially altered by [K^+^].

*CR*_w-i_ values for the mutant were greater than those for the wild-type over the entire concentration range, suggesting that fewer K^+^ ions occupied the selectivity filter in the mutant. These results demonstrate that blocking the low-affinity site in the cytoplasmic pore significantly modifies the permeation mode in the selectivity filter.

### A permeation model for the Kir2.1 channel

To investigate how the inward rectification of the E224K/H226E mutant involves modified permeation in the selectivity filter, we built a simple model for the Kir2.1 channel. Permeation processes through the wide-pore region, including transitions from the cytoplasmic pore towards the central cavity, must be involved in the permeation model. For simplicity, we assign a single K^+^ binding site in the wide pore, even if this long and wide pore may have multiple K^+^ binding sites. This site is named the wide-pore K^+^ binding site, which is a lumped K^+^ binding site. Because this site is located outside the membrane electric field, the rate constants for the binding and release of K^+^ from the intracellular bulk solution to the wide-pore K^+^-binding site were set to be voltage-independent. In contrast, the binding of K^+^ to this site should be affected by the binding of polyamine to the low-affinity binding site via an electrostatic effect. A simple eleven-state model was generated to characterize the Kir2.1 channel (Fig. 6a; see Methods for details). Given a set of rate constants, ion flux and *CR*_w-i_ were calculated with the cycle flux algebra (see Methods)^41^,^42^.

First, rate constants were optimized for the wild-type channel via the experimental data of both current-voltage and *CR*_w-i_−[K^+^] curves (see Methods). As shown in the previous paper^30^, *CR*_w-i_ values serve as effective constraints for the optimization of rate constants. Both current-voltage and *CR*_w-i_−[K^+^] curves calculated from the optimized rate constants were superimposed on experimental data (solid lines in Figs 2b,c and 5d). The model reproduced linear current-voltage relationships at high [K^+^] and substantial changes in *CR*_w-i_ values in the [K^+^] range tested. Unlike the fit of the Michaelis-Menten equation to the data, the model predicted that there were two phases in the concentration dependence of current amplitudes (Fig. 2c, solid lines).

In the mutant channel, the wide-pore K^+^-binding site is strongly modified by the mutation E224K/H226E, which should have a substantial consequence for changes in the rate constants related to the binding and release of K^+^ in the wide-pore binding sites. Thus, the modeling for the mutant channel was first performed by assuming that the effect of the mutation is limited to the process of K^+^ binding and release to and from the wide-pore K^+^-binding site. The related rate constants involve the binding (k1) and releasing (k2) rate constants as well as the rate constant for the transition between the wide-pore K^+^-binding site and the selectivity filter (k5 for a one-ion occupied selectivity filter; k7 and k8 for a double-ion occupied selectivity filter) (Fig. 6a). The optimization was performed for these five rate constants, while the rest of rate constants were fixed as those of the wild-type channel (see Methods for details). The current−voltage and *CR*_w-i_−[K^+^] curves were superimposed on experimental data for the mutant [Figs 2b,c (solid line) and 5d]. The parameter optimizations were also performed with smaller (four free parameters) and larger (ten free parameters) numbers of free-rate constants, and the fitting was evaluated with the Akaike Information Criterion (AIC; see Methods)^43^. The AIC values for the four-, five- and ten-parameter fit were 3.45, 2.98 and 4.90, respectively, and the model of five free-rate constants statistically generated the best fit to the data.

The modified rate constants (k1, k2, k5, k7 and k8) are shown on the permeation diagram (Fig. 6b), in which relative changes in rate constants are expressed with solid (increased) or broken (decreased) arrows. The absolute rate constants for the wild-type and mutant channels are shown in the Supplementary Information (Supplementary Fig. S1). The increased rate of binding to the wide-pore K^+^-binding site (k1) and the decreased rate of release (k2) indicate that the affinity of K^+^ increased rather than decreased in the mutant channel. In contrast, the rates for the ion transfer from the wide-pore K^+^-binding site to the selectivity filter (k5 and k7) were substantially attenuated. These results were not expected in the previous papers^17^,^18^,^19^,^20^.

### Visualization of the permeation process

The permeation feature of the model was visualized by inspecting the contribution of cyclic paths on the model diagram (Fig. 6a right panel, also see Methods and Supplementary Information). A net ion flux was decomposed into cycle flux of each cyclic path, *i* (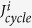), which carries a defined ratio between water and ion fluxes (the cyclic coupling ratio of water and ions, *ccr*_w-i_). For example, starting from state 1, a transition follows a route such as 1 → 2 → 3 → 1 and generates a cycle “***a***” (red cycle, Fig. 6a, right panel). The arrangement of water molecules and ions in the selectivity filter is i-w-i-w in state 1, i-w-i-w in state 2, and w-i-w-i in state 3. In addition, one ion occupies the wide-pore binding site in state 2. Upon the completion of cycle ***a***, one water molecule and one ion are transferred, and the *ccr*_w-i_ value for cycle ***a*** is one. Among the cycles, there are low-profile paths having lower barriers (and higher rate constants), and cyclic transitions through these paths give higher cycle flux and are more frequently used than other cycles.

The cycle flux algebra (Methods) demonstrates that there are 39 cyclic paths with non-zero flux in the diagram, but the fitted model revealed that only a few of them with short-path cycles, such as cycles ***a, b***and***c***, contributed predominantly to the total net ion flux in both the wild-type and E224K/H226E mutant (Fig. 7a). Supplementary Fig. S2 shows the relative cycle contributions at different membrane potentials at 150 mM [K^+^]. The relative contributions of cycles to total flux (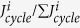) were plotted as the heights of cyclic paths raised from the bottom, where the permeation diagram was shown (footprint). The contributions of both cycles ***a*** and ***b*** decreased, whereas that of cycle ***c*** increased as the membrane potential became more positive in the wild-type channel. The conductance of each individual cycle (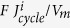; where *F* is the Faraday constant) and the total conductance were plotted against voltages for wild-type and mutant channels (Fig. 7a). In the wild-type, the total conductance was nearly constant, whereas the conductance of each individual cycle varied substantially in the voltage range tested. As the membrane potential became more positive, the contributions of both cycles ***a*** and ***b*** decreased, whereas those of cycles ***c, d***and***e*** increased. These changes in cycles compensated each other and produced a nearly constant conductance, i.e., a linear current-voltage curve. In the E224K/H226E mutant, the predominant cycles were cycles ***a***–***c***. Of note, the conductance contribution of cycle ***a*** decreased substantially due to the modification of rate constants by mutations, whereas the conductance from cycles ***b***and ***c*** were similar to those in the wild type. The dramatic decrease in the conductance from cycle ***a*** resulted in attenuated total currents, especially at positive potentials, and thereby a reduced single-channel conductance and an inward rectification of the current-voltage curve (Fig. 2).

Figure 7b shows the effects of [K^+^] on relative cycle contributions to total conductance. At high [K^+^], cycle **a** predominantly contributed to total flux, but flux contributions from cycles ***c***, ***d***, and ***e*** whose *CR*_w-i_ values are two, three and three, respectively, became more important at low [K^+^] in the wild-type channel. Even if the involved cycles were changing and thus *CR*_w-i_ changed with [K^+^], the sum of the flux was similar at different [K^+^]. These features explain why the conductance saturates (Fig. 2b) but *CR*_w-i_ remains varying at the [K^+^] range tested (Fig. 5d). In the E224K/H226E mutant, at high [K^+^], cycle ***b*** predominantly contributed to the total conductance, but flux from cycle ***c*** with a *CR*_w-i_ value of two took the lead at low [K^+^]. Despite the preferred cycles were different at varying [K^+^], the total cycle flux remains about the same, and thus the conductance saturates at low [K^+^] in a way similar to the wild-type channel. Note that in the E224K/H226E mutant, as [K^+^] was increased, the contribution of cycle ***c*** became less pronounced and that of cycle ***b*** increased. In other word, the predominant flux cycles involved both one-ion and two-ion modes in the selectivity filter. This explains why the averaged flux ratio was around 1.5 in the E224K/H226E mutant at physiological [K^+^] (150 mM) (Fig. 5d). It is noted that Fig. 7b shows the relative contribution of each cycle flux to total flux. The conductance of each cycle also depends on rate constants involved in the cyclic paths (Eq (3) in Methods). For example, although the relative contribution of cycle c to total flux was higher in the mutant (Fig. 7b), the conductance of cycle c was about the same in both wild-type and mutant channels (Fig. 7a) due to modification of rate constants by mutations.

In summary, data and model analyses suggest that the dramatic attenuation of outward currents in the E224K/H226E mutant is explained by the permeation through the less ion-occupied selectivity filter, which can be accounted for by modifying the rate constants that are relevant to the binding and release to and from the wide-pore K^+^-binding site. In addition, the attenuated rates for transitions between the cytoplasmic pore and selectivity filter resulted in the reduced contribution of cycle ***a*** to total conductance, especially at positive potentials, thereby resulting in attenuated outward currents.

## Discussion

In this study, we examined the mechanism underlying altered permeation properties in the E224K/H226E mutant, in which a positive charge is added to the low-affinity blocking site (E224). This mutant allows us to measure reversal potentials that cannot be evaluated in the presence of blockers and thus to estimate V_stream_. In single-channel recordings, we revealed that the voltage-dependent attenuation of macroscopic outward currents can be attributed to the inward-rectified permeation features of the E224K/H226E mutant.

To assess the mechanism underlying the permeation alteration by mutation, [K^+^]-dependent *CR*_w-i_ values were evaluated from measurements of V_stream_ and single-channel conductance at different [K^+^]. As we have shown in a previous paper^30^, the [K^+^]-dependent *CR*_w-i_ values provide intuitive clues concerning which cyclic paths in the permeation diagram are predominantly used in a given condition. We exploited *CR*_w-i_ values and single-channel currents to envision the dynamic processes underlying ion permeation in Kir2.1 channels using the cycle flux algebra.

**Figure 1:**
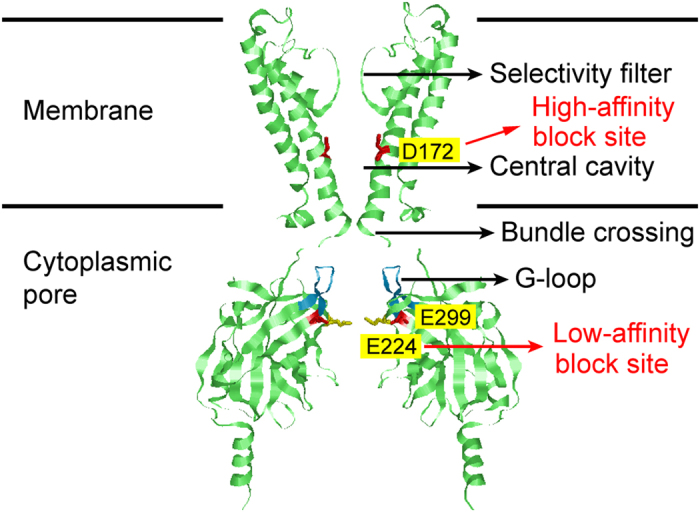
Homology model of Kir2.1 channels. (**A**) Construction of the model was based on a sequence alignment with the structure of a Kir2.2 channel^51^. Two of the four subunits of the Kir2.1 channel are shown. The channel pore consists of the indicated selectivity filter, central cavity and cytoplasmic pore. Residues involved in polyamine binding are shown in ball-and-chain models and are highlighted by yellow markers.

Previous studies have demonstrated that negative charges in the intracellular pores enhance channel conductance in several types of K^+^ channels, presumably by concentrating local [K^+^]^18^,^22^,^23^,^24^. The binding of positively charged polyamines to glutamate residues in the cytoplasmic pore may reduce K^+^ occupancy. In addition, experimental evidence indicates that the binding of polyamines at the low-affinity site can produce a narrowing effect at the bundle crossing (Fig. 1) and thus reduce the single-channel conductance in Kir2.1 channels^18^,^19^, which suggests an increase in the energy barrier for intracellular K^+^ entering the central cavity. Recently, it has been proposed that the peak of this energy barrier for both intracellular permeate ion and blocker is very close to the inner end of the bundle crossing in terms of electrical distance (δ ≈ 0)^44^. However, our data and model analysis provide an alternative mechanism for events occur at the cytoplasmic pore of the Kir2.1 channel.

Our data and model analyses reveal that the rate constants related to binding to the wide-pore K^+^ binding site and those related to transfer from the site to the selectivity filter in the mutant were modified from those of the wild-type channel. These results indicate that the mutational effects are not confined to the wide-pore binding site but extend to ion permeation processes in the selectivity filter, which are under the strong influence of an electrical field. Accordingly, current-voltage curves became inwardly-rectifying in the mutant.

**Figure 2:**
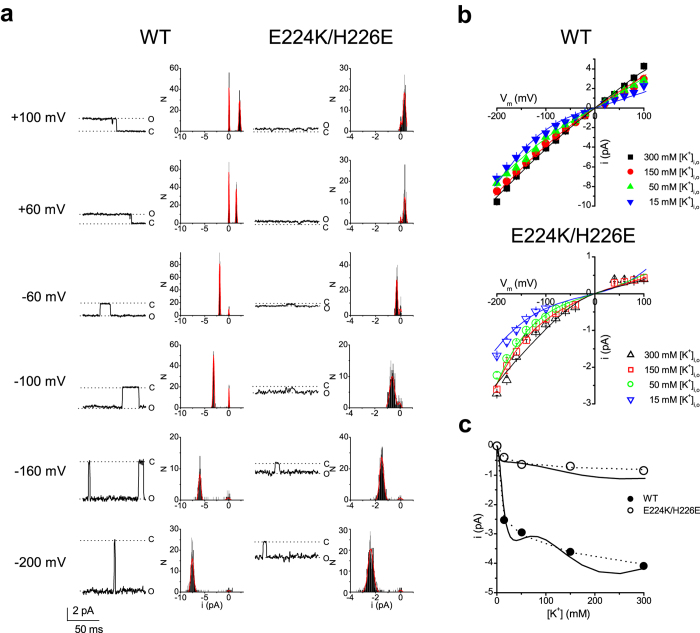
Comparison of single-channel conductance in wild-type Kir2.1 channels and E224K/H226E mutants. (**a)** Current traces and the all-point-histograms of the wild-type and the E224K/H226E mutant at the indicated voltages. At all of the voltages tested, the single-channel current (i, left panels) was smaller in the mutant than in the wild-type channel. However, the probability of the opening of the E224K/H226E channel (right panels) did not seem to be different from that of the wild-type channel. (**b)** Single-channel current−voltage (i-V_m_) relationships at varying K^+^ concentrations. At low K^+^ concentrations, the current−voltage curve displays strong inward rectification in the E224K/H226 mutant and mild inward rectification in the wild-type. Symbols (squares, circles and triangles) represent experimental data, and the lines depict the model’s fit to the data. n = 2–6 for both the wild-type and mutant. (**c**) The [K^+^]-dependence of the single-channel conductance at −100 mV. The broken lines and solid lines are the fit of the Michaelis-Menten equation and the permeation model (Fig. 6), respectively, to the data with the value at 300 mM as the maximum.

**Figure 3:**
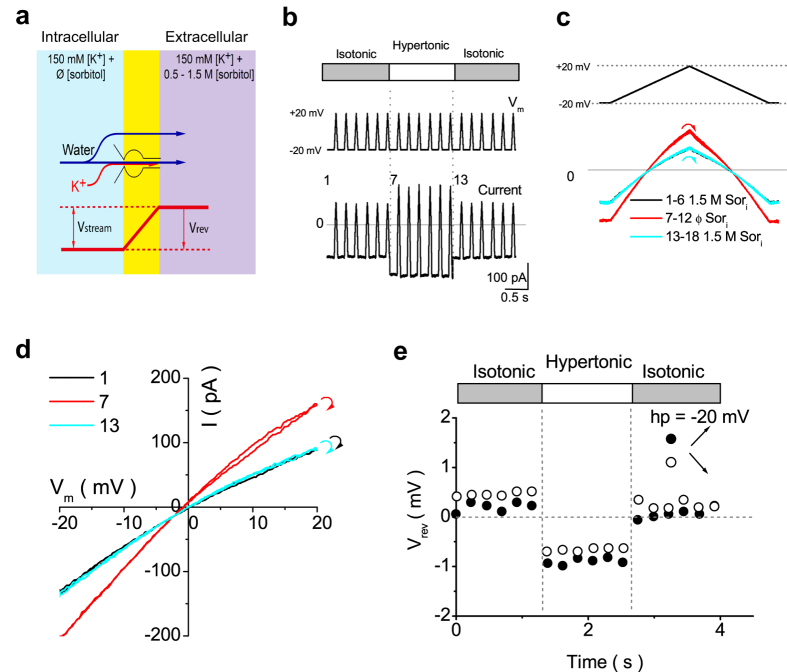
Measurements of V_stream_ in the wild-type Kir2.1 channel. (**a)** The experimental configuration of the coupled water-K^+^ movement and the resultant negative shift of V_rev_ when the intracellular osmotic pressure is lower than the extracellular osmotic pressure. (**b)** Voltage protocol and a set of representative current traces. From a holding potential (hp) of −20 mV, a train of ramp voltage (−20 to +20 mV, rate of change 1 mV/ms) was applied before, during, and after the osmotic pulse. (**c)** The enlargement of a ramp set (positive and negative ramps) and current traces. Currents recorded under the same intracellular sorbitol concentration (Sor_i_) overlapped with one another. The current−voltage curves obtained from the negative and positive ramps were nearly identical to each other, and the two traces crossed at the same V_rev_. (**d)** Current−voltage relationships (of both ramp branches) at ramp 1, 7 and 13. The curved arrows at the end of the data at +20 mV indicate the transition from the positive to negative ramp. **e**. The time course of V_rev_. Filled and open circles denote data obtained from the positive (●) and negative ramps (○), respectively. The average V_stream_ values are −1.57± 0.26 mV at 15 mM [K^+^], with ΔOsm = 1.5 M.

**Figure 4:**
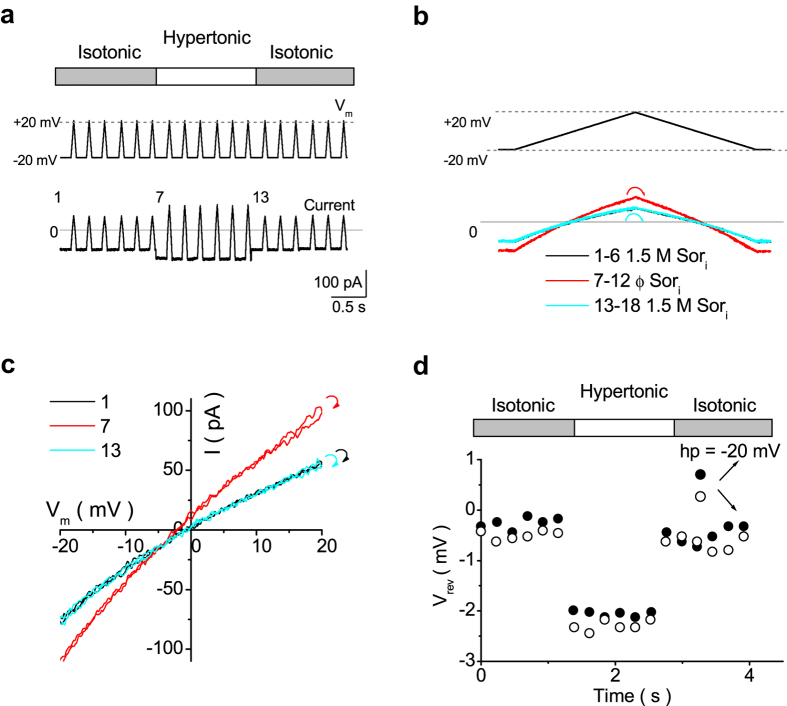
Measurement of V_stream_ in the E224K/H226E mutant. (**a**) Ramp protocol and a set of representative current traces. (**b)** The enlargement of a ramp set and current traces. Currents recorded under the same intracellular sorbitol concentration (Sor_i_) overlapped one another. (**c)** Current−voltage relationships (of both ramp branches) at ramps 1, 7 and 13. The curved arrows at the end of the data at +20 mV indicate the transitions from the positive to negative ramp. (**d)** The time course of V_rev_. Filled and open circles denote data obtained from the positive (●) and negative ramps (○).

The affinity of K^+^ to the wide-pore binding site was enhanced in the mutant, and bound K^+^ cannot readily pass the high barrier to enter the selectivity filter, leading to attenuated outward currents. Thus, in this model, the apparent K_m_ value for the mutant estimated from the concentration-dependent current amplitude (Fig. 2c, broken lines by the Michaelis-Menten fit) do not reflect the affinity of K^+^ to the wide-pore K^+^ binding site. Instead, the wide-pore binding site is nearly saturated at a low K^+^ concentration (the first phase predicted by the model, solid line, Fig. 2c); thus, the processes following the binding, such as transfers from the binding site to the selectivity filter, determine the permeation rate (the second component).

Why does the affinity for K^+^ increase in the wide pore even under the influence of the electrostatic repulsive effect in the E224K/H226E mutant? In the model, we introduced a K^+^ binding site in the wide pore to the canonical permeation model proposed by Morais-Cabral *et al.*^25^. For simplicity, only one K^+^ binding site was introduced along the long, wide pore. Adding additional K^+^ binding sites makes the cycle flux calculation too complicated to be carried out. Thus, for the modeling, we take the conventional approach of lumping multiple sites into one. In our model, the wide-pore binding site represents all possible bindings along the long pore instead of the one at site 224 and the Km value represents overall binding characteristics. For example, when the affinity for K^+^ is decreased at one of the multiple sites, K^+^ affinity may increase in other binding sites in the wide pore. It is also plausible that the alternative site may have a higher affinity because of attenuated repulsive force caused by K^+^ binding to the previous site. These hypothetical issues should be examined in the future by focusing on permeation process occurred the wide pore.

## Conclusion

In ion channels, water flux is accompanied by ion conduction at a comparable rate^45^. This water flux is mandatory in a narrow pore where ions and water molecules form a single file, and we exploited this phenomenon to envision dynamic processes underlying ion permeation in Kir2.1 channels. Several studies have shown that the electrostatic characteristics of the cytoplasmic pore affect ion conductance in ion channels^18^,^22^,^23^,^24^. This effect has been explained primarily by local ion-concentrating mechanisms and energy barrier features. In this study, we obtained experimental evidence that changes of the electrostatics at the low-affinity site of the Kir2.1 channel affects permeation processes in the selectivity filter. Because electrostatic effects on channel conduction are prevalent, the mechanism proposed in this study may have broad usefulness in understanding the permeation of ion channels and the patho-physiological functions of cells employing ions for signal transduction.

## Methods

### Preparation of Xenopus oocytes and generation of E224K/H226E mutants

Oocytes were isolated using a partial ovariectomy of Xenopus laevis (South Africa) anesthetized with 0.1% (w/v) tricaine (3-aminobenzoic acid ethyl ester), as previously described^46^. The surgical protocol was conducted in accordance with the Guide for the Care and Use of Laboratory Animals (1996, National Academy of Sciences, Washington, D.C.). Oocytes were maintained at 18 °C in Barth’s solution (pH 7.6) containing NaCl 88 mM, KCl 1 mM, NaHCO_3_ 2.4 mM, Ca(NO_3_)_2_ 0.3 mM, CaCl_2_ 0.41 mM, MgSO_4_ 0.82 mM, HEPES 15 mM and gentamicin 20 μg/ml. E224K/H226E mutants were generated using polymerase chain reactions, and the correctness of the mutations was confirmed by the sequencing of the cDNAs using an ABI Prism^TM^ dRhodamine Terminator Cycle Sequencing Ready Reaction Kit (PE Applied Biosystems, Foster City, CA). cRNAs were obtained using *in vitro* transcription (mMessage mMachine, Ambion, Dallas, USA) and were injected into *Xenopus* oocytes (60−125 ng for giant-patch experiments and 0.03−0.3 ng for single-channel recordings) to be used 1–3 days after the cRNA injection.

### Electrophysiological recordings

Currents were recorded at room temperature (21−24 °C) using patch-clamp techniques 47. The symmetrical 15 to 300 mM [K^+^] solution (pH 7.4) contained 9.15−294.15 mM KCl, 3.6 mM KOH, 1 mM EDTA, 1 mM K_2_HPO_4_, and 0.25 mM KH_2_PO_4_. Sorbitol (0.5−1.5 M) was added to the solution for the osmotic pressure experiments. To accurately estimate the reversal potentials, we first carried out capacitance and series compensation (80% compensation) online. The residual capacitive current was eliminated by subtracting the currents recorded before the channel rundown from those recorded after the complete rundown (Supplementary Fig. S3). The sampling/filtering frequencies were 10 kHz/1 kHz for the giant-patch recordings and 5 kHz/1 kHz for the single-channel recordings. Command voltages were controlled by and data acquired with pClamp10 software (Molecular Devices).

### Measurement of V_stream_

V_stream_ was measured as previously described^30^,^31^. Briefly, V_stream_ was estimated as the change of V_rev_ upon the establishment of an osmotic gradient across the membrane. To apply osmotic pulses, we used a double-barrel tube controlled by a rapid solution-exchange system (SF-77B perfusion system, Warner Instruments, Hamden, USA), which in turn was controlled by pClamp10. The reference electrode (3 M KCl) was placed adjacent and downstream to the patch electrode during the osmotic pulse experiment to avoid establishing a liquid junction potential between the solution during the osmotic pulse and the bath solution. The liquid junction potentials of the reference electrode in the normal- and hyper-osmotic solutions were measured (Table 1) and corrected^30^. Further, voltage drifts were not detected during the short recording period.

The osmolarities of solutions were measured using an osmometer (Orion Star A214, Thermo Scientific, Waltham, USA) and adjusted to desired values by adding sorbitol. To maintain constant K^+^ activities in the absence and presence of sorbitol at a constant [K^+^], solutions with and without sorbitol were adjusted to the same [K^+^] as measured with a K^+^-selective electrode (Orion 97-19 Ionplus Potassium Electrode; Thermo Electron Corporation, Madison, USA).

### Permeation model

The permeation model (discrete-state Markov model) involving a K^+^ binding site in the cytoplasmic pore was constructed based on a model previously used for potassium channels (the canonical model)^30^,^48^,^49^. In the model, the rate constant *k*_i_ is defined as follows:


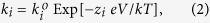


where *k*^o^ is the rate constant at 0 mV and *z*_i_ is the electrical distance. In the previous canonical model^25^, either an ion or a water molecule occupies one of the four binding sites in the selective filter, and two ions are not allowed to occupy adjacent positions because of electrostatic repulsion, which results in eight ion distribution states (the eight-state canonical model). In the present model, an ion-empty state was eliminated because none of the experiments were performed at extremely low K^+^ concentrations. The voltage dependency of rate constants and degenerated rate constants were set identical to that of the canonical model proposed by Morais-Cabral *et al.*

An additional cytoplasmic pore K^+^ binding site was introduced as a voltage-independent binding site because the site is located outside the membrane electric field (Fig. 6a, left). Thus, the rate constants for both the binding and release of K^+^ from the intracellular bulk solution to the cytoplasmic pore site were set to be voltage-independent (z = 0). The procedure of model development is shown in Supplementary Fig. S4. First, the canonical model was doubled such that the wide pore has either one or no ion. These two models were then verged with extra transition paths. The model is further simplified by deleting similar ion-occupying states and the related transitions.

By introducing the cytoplasmic-pore K^+^-binding site, the number of states in the model should be doubled, but three of the cytoplasmic pore-bound states were pruned such that the overall permeation routes on the state diagram were not perturbed. By reducing the number of states, the subsequent cycle flux algebra was dramatically simplified. Accordingly, an eleven-state permeation model characterizing permeation processes through the cytoplasmic pore and the selectivity filter with minimal complexity was formed.

On this permeation diagram, the number of rate constants should be 34, but thermodynamic cyclic reversibility reduces the number of free parameters^50^. For example, the rate constant for a transition from state 3 to 2 (*k*_32_) was calculated as 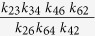. Moreover, rate constants sharing similar physical processes, such as the binding to the cytoplasmic pore site, could be degenerated. For example, in all of the transitions 1 → 2, 5 → 6, and 10 → 11, intracellular K^+^ binds to the cytoplasmic pore site; thus, these rate constants were set identical (degenerated rate constants). The *z*_i_ values of rate constants were fixed in a manner similar to that used in the previous model, implementing the location of ion binding sites in the electric field^30^,^48^. Except for the rate constants of K^+^ association and dissociation between the intracellular solution and the cytoplasmic pore site (*z*_1_ = 0 and *z*_2_ = 0), the z values for the other rate constants were defined as non-zero values: *z*_3_ = 0.2, *z*_4_ = −0.2, *z*_5_ = 0.3, *z*_6_ = 0.2, *z*_7_ = 0.2, *z*_8_ = −0.2, *z*_9_ = 0.1, *z*_10_ = −0.1. With these procedures, the number of free parameters in this model became ten, which were optimized. The set of rate constants after degeneration is shown on transition paths in the state diagram (Fig. 6a, left).

### Cycle flux algebra

The ion flux generated in the model at a given [K^+^] and voltage were calculated using cycle flux algebra, which gives exact solutions for ion flux^42^, rather than relying on approximated values from a Monte Carlo simulation.

The methods for a graphical solution of one-way cycle flux and numerical evaluations were performed as previously described^30^,^42^. Additionally, this calculation gives *CR*_w-i_ values because each cycle carries a defined ratio of ion to water fluxes (*ccr*_w-i_) as follows. The net ion flux is decomposed into fluxes through cyclic paths on the diagram (i.e., the cycle flux, 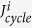; the number of ions transferred per second on a given cyclic path, *i*; Fig. 6). The cycle fluxes for all possible cyclic paths on the diagram are calculated from an integration of rate constants. Each cyclic path carries not only an ion flux but also a water flux, and the ratio between water and ion fluxes is intrinsically defined based on the detailed permeation process through relevant cycles. This ratio is defined as the cyclic coupling ratio, 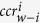 (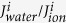
for cycle *i*), which is similar to the stoichiometric number of chemical reactions. For example, starting from state 1, a transition follows a route such as 1 → 2 → 3 → 1 and generates a cycle ***a*** (red cycle, Fig. 6a, right panel). The arrangement of water molecules and ions in the selectivity filter is i-w-i-w in state 1, i-w-i-w in state 2, and w-i-w-i in state 3. In addition, one ion occupies the cytoplasmic site in state 2. Upon the completion of cycle ***a***, one water molecule and one ion are transferred, so 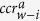 is one (Fig. 6). Taking another example, cycle ***c*** (4 → 6 → 11 → 9 → 7 → 4) transfers two water molecules and one ion, so 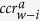 is two. Thus, the experimentally obtained *CR*_w-i_ value is the weighted sum of 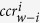 (Eq.).


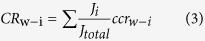


where J_i_/J_total_ is the relative contribution of cycle *i* to the total flux. If a measured CR_w-i_ value is 1.5, then cycle ***a***, cycle ***c***, and possibly other cycles are contributing.

As the number of states increases, combinatorial calculations in the cycle flux algebra increase dramatically, while the eleven-state model was feasible for the cycle flux algebra and the numerical calculations within practical time. The state diagram was graphically decomposed into sub-states having one-way cyclic paths. There are altogether 231 sub-states. Inversion of the sub-state matrix with dimensions of 231 × 231 gave the steady-state probability of sub-states, from which a one-way cycle flux was calculated. There are 59 cyclic paths, each giving defined ion and water fluxes, and 39 of them yield non-zero cycle fluxes.

### Parameter optimization

**Figure 5:**
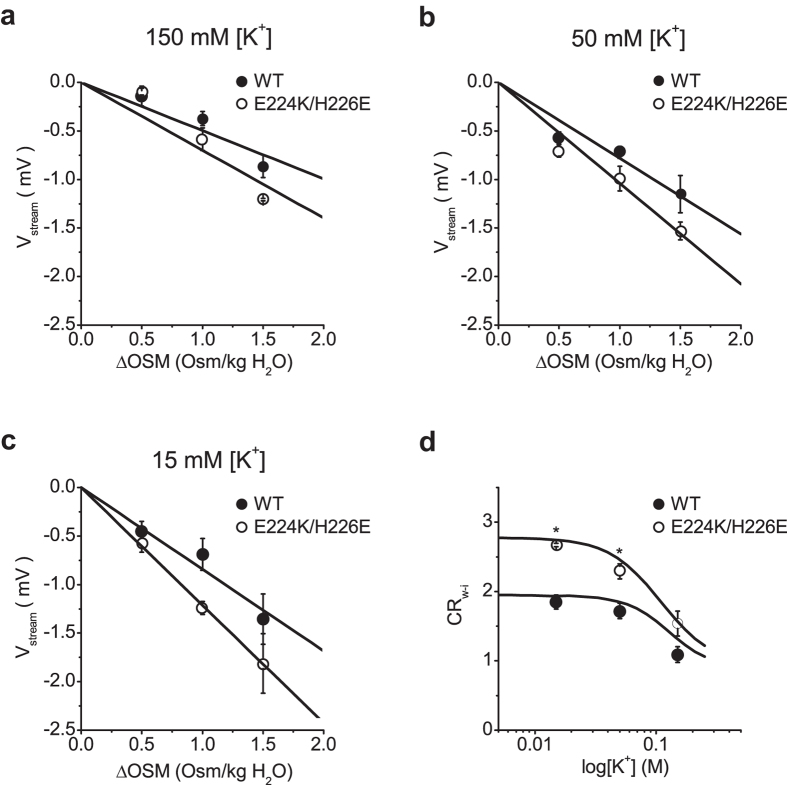
Dependence of V_stream_ on ΔOsm and [K^+^]. (**a)** The two V_stream_ – ΔOsm relationships were linear, with slope values of −0.63 ± 0.02 (mean ± standard error) and −0.83 ± 0.07 mV/Osm/kg in the wild-type and the mutant, respectively, at 150 mM [K^+^]. The line appeared to be steeper in the mutant than in the wild type, although there was no significant difference between the two regression lines (p = 0.07). (**b)** At 50 mM [K^+^], the two slope values were significantly different, with −0.82 ± 0.01 in the wild-type and −1.08 ± 0.02 mV/Osm/kg in the mutant (p < 0.05). (**c)** At 15 mM [K^+^], the slope value further increased, to −0.98 ± 0.07 and −1.35 ± 0.03 mV/Osm/kg for the wild-type and the mutant, respectively. The two slopes were significantly different (p < 0.05). (**d**) The relationship between CR_w-i_ and log[K^+^]. The CR_w-i_ value decreased from 2.15 ± 0.15 at 15 mM [K^+^] to 1.39 ± 0.04 at 150 mM [K^+^] (1.80 ± 0.03 at 50 mM [K^+^]) for the wild-type channel. The CR_w-i_ values for the E224K/H226E mutant changed from 2.98 ± 0.07 at 15 mM [K^+^] to 1.84 ± 0.15 at 150 mM [K^+^] (2.37 ± 0.04 at 50 mM [K^+^]). The black curves were calculated from the model using cycle flux algebra. n = 3 to 6 for wild type; 3 to 4 for E224K/H226E.

**Figure 6:**
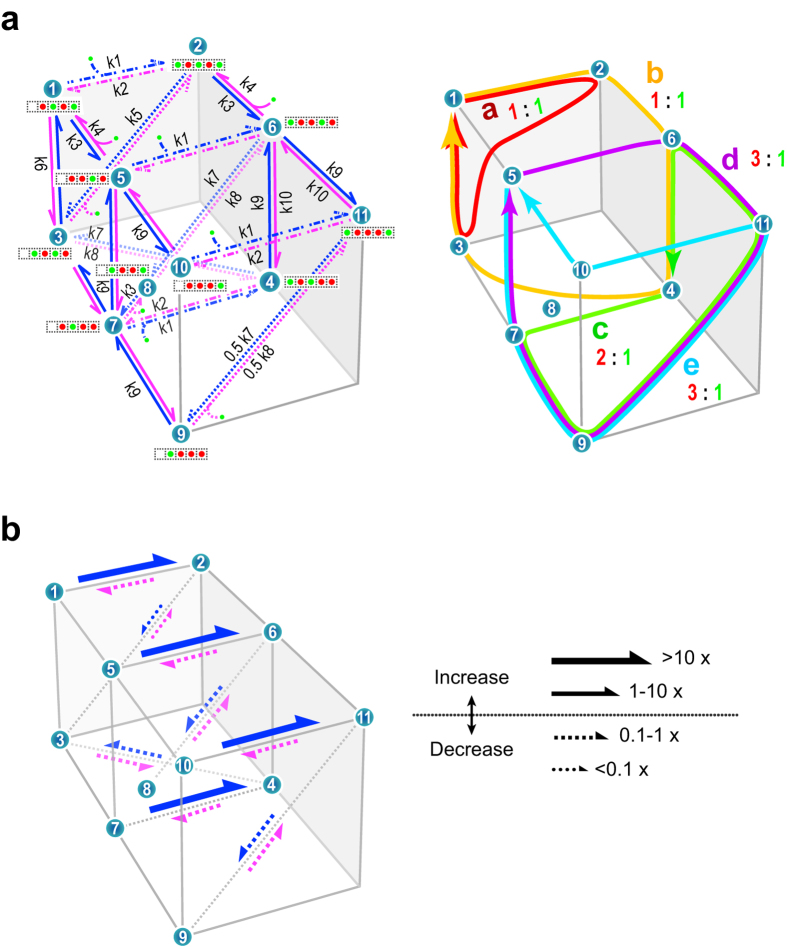
Permeation model, parameters and permeation features for the wild-type and mutant channels. (**a)** Left panel: The eleven-state permeation diagram for K^+^ (green circle) and water (red circle) permeation through a Kir2.1 channel (left side, intracellular). Each green channel cartoon represents a K^+^-water-occupied state in the selectivity filter and cytoplasmic pore. Arrows denote states transitions, which accompany shift movements of K^+^-water columns. Blue and magenta arrows indicate transitions for the efflux and influx, respectively. Curved lines represent K^+^ entering from either side to the cavity or the selectivity filter, while curved arrows represent K^+^ exiting from them. k1 through k10 represent the rate constants for the state transitions. Right panel: Cyclic paths on the permeation diagram. Each cycle was drawn by connecting states, and by completing a cycle, a net transport of K^+^ and water occurred. In each cycle, the ratio of the coupled movements of water (red number) and K^+^ (green number) molecules is indicated. For example, cycle ***a*** exhibits a coupling ratio of 1:1 and cycle ***c***, 2:1. (**b)** The affected path and the degree of modification of rate constants in the mutant channel. Solid arrows indicate transition paths with increased rate constants, whereas broken arrows indicate those with decreased rate constants.

**Figure 7:**
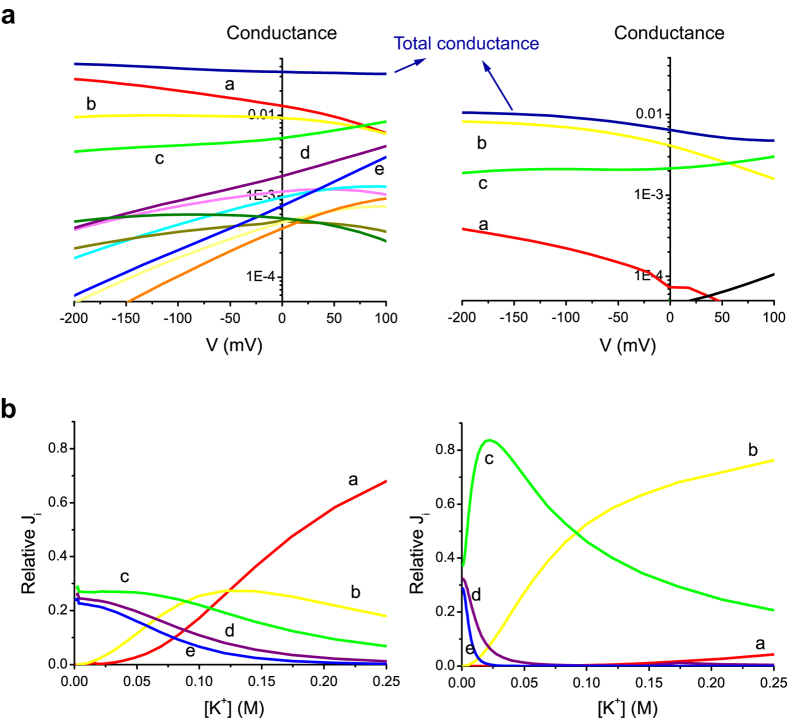
Permeation features for the wild-type and mutant channels. (**a**) The conductance of the wild-type (left) and mutant (right) channels as a function of the membrane potential at 150 mM K^+^. The total conductance is nearly flat for the wild-type channel, but it is attenuated at the positive potentials or inward-rectified for the mutant channel. In the wild-type channel, the underlying conductances of the cycle fluxes indicate that the voltage-dependent increasing (cycle ***c***) and decreasing (cycle ***a*** and ***b***) cycles compensate for yielding a nearly constant conductance. In the mutant channel, cycle ***a*** is attenuated substantially, and the remaining cycles ***b*** and ***c*** generate attenuated conductance at positive potentials. (**b)** The relative flux contributions to conductance by various cycles in the wild-type (left) and mutant (right) channels at various [K^+^].

The experimental results of current–voltage and *CR*_w-i_–[K^+^] curves were used to optimize rate constants for the wild-type channel. Parameter optimization was performed using the Nelder-Mead algorithm. The number of data for *CR*_w-i_–[K^+^] curves was much smaller than that of current–voltage curves, and thus, the former data were weighted for the evaluation of goodness-of-fit. Repeated optimizations with varying weights led to the parameters that best fit the data. After thoroughly searching, the model with optimized parameters successfully reproduced current–voltage and *CR*_w-i_–[K^+^] curves for the wild-type channel (Figs 2b and 5d).

For the E224K/H226E mutant, optimizations were performed for different numbers of free parameters. Starting from four free parameters related to the cytoplasmic K^+^ binding site (k1, k2, k5 and k7), the number of the free parameters was increased to five (k1, k2, k5, k7 and k8), involving permeation processes across the cytoplasmic pore towards the central cavity. In these cases, the other rate constants were fixed to the wild-type values (limited parameter fitting). Additionally, all ten rate constants were set free and then optimized for the mutant. Among the three optimizations that had four, five and ten free parameters, statistical evaluations were performed to select the valid model by using the Akaike information criterion (AIC), in which the maximal likelihood values (L) as well as the number of free parameters (k) are considered for the best parameter set as follows.


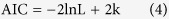


Mathematica ver. 10 (Wolfram Research, Champaign, USA) was used for all graphical and numerical calculations as well as numerical optimizations.

### Data analysis

Averaged data are presented as means ± SE. Analysis of covariance was used to test whether two regression lines were significantly different.

## Additional Information

**How to cite this article**: Chang, H.-K. *et al.* Mechanism for attenuated outward conductance induced by mutations in the cytoplasmic pore of Kir2.1 channels. *Sci. Rep.* **5**, 18404; doi: 10.1038/srep18404 (2015).

## Supplementary Material

Supplementary Information

## Figures and Tables

**Table 1:** Liquid junction potentials of the reference electrode (3M KCl) between the normal- and hyper-osmotic solutions.

	0.5 M Sorbitol	1.0 M Sorbitol	1.5 M Sorbitol
15 mM [K^+^]	−0.22 ± 0.199 mV	−0.14 ± 0.084 mV	−0.03 ± 0.038 mV
50 mM [K^+^]	−0.06 ± 0.022 mV	−0.12 ± 0.028 mV	0.13 ± 0.054 mV
150 mM [K^+^]	−0.12 ± 0.012 mV	−0.22 ± 0.043 mV	−0.17 ± 0.057 mV
