# Supplementary material for: Mechanism for attenuated outward conductance induced by mutations in the cytoplasmic pore of Kir2.1 channels
Source: Sci Rep. 2015 Dec 18;5:18404. doi: 10.1038/srep18404 (PMC4683409; doi:10.1038/srep18404)
Supplement: Supplementary Information [file srep18404-s1.pdf]

Supplementary Information

Mechanism for attenuated outward conductance induced by mutations in the cytoplasmic pore of Kir2.1 channels

Hsueh-Kai Chang[1], Masayuki Iwamoto[2], Shigetoshi Oiki[2#], and Ru-Chi Shieh[1#]

[1]Institute of Biomedical Sciences, Academia Sinica, Taipei 11529, Taiwan, ROC

[2]Department of Molecular Physiology and Biophysics, University of Fukui Faculty of Medical Sciences Eiheiji-cho, Yoshida-gun, Fukui 910-1193, Japan

Parameter fitting for the wild-type and mutant channels

For the wild-type channel, optimization of the ten free parameters were performed repeatedly from systematic changes for the initial parameter sets.

Fitted rate constants for the wild-type are (/ms): $k_{12} = 1.27 \times 10^7$, $k_{23} = 1.59 \times 10^5$, $k_{31} = 1.86 \times 10^9$, $k_{34} = 1.55 \times 10^9$, $k_{15} = 2.29 \times 10^6$, $k_{46} = 9.76 \times 10^6$, $k_{26} = 2.29 \times 10^6$, $k_{56} = 1.27 \times 10^7$, $k_{57} = 1.08 \times 10^7$, $k_{47} = 1.28 \times 10^5$, $k_{78} = 3.44 \times 10^3$, $k_{86} = 1.55 \times 10^9$, $k_{79} = 5.89 \times 10^5$, $k_{510} = 9.76 \times 10^6$, $k_{611} = 9.76 \times 10^6$, $k_{911} = 1.74 \times 10^7$, $k_{1011} = 1.27 \times 10^7$, $k_{21} = 1.28 \times 10^5$, $k_{32} = 2.05 \times 10^9$, $k_{13} = 1.43 \times 10^7$, $k_{43} = 2.60 \times 10^4$, $k_{51} = 5.44 \times 10^5$, $k_{64} = 1.08 \times 10^7$, $k_{62} = 5.44 \times 10^5$, $k_{65} = 1.28 \times 10^5$, $k_{75} = 9.76 \times 10^6$, $k_{74} = 1.27 \times 10^7$, $k_{87} = 2.29 \times 10^6$, $k_{68} = 2.60 \times 10^4$, $k_{97} = 9.76 \times 10^6$, $k_{105} = 1.08 \times 10^7$, $k_{116} = 1.08 \times 10^7$, $k_{119} = 1.30 \times 10^4$, $k_{1110} = 1.28 \times 10^5$.

For the mutant channel, as shown in the Method section, three different optimizations having different numbers of free parameters were performed, and the results were evaluated with the AIC as follows. The AIC value was 5.1, 2.8, and 9.8 for the four, five and ten parameter fit, respectively. Thus, we selected the five parameter model for the mutant channel.

For the mutant: $k_{12} = 4.73 \times 10^9$, $k_{23} = 3.59 \times 10^2$, $k_{31} = 1.86 \times 10^9$, $k_{34} = 3.40 \times 10^8$, $k_{15} = 2.29 \times 10^6$, $k_{46} = 9.76 \times 10^6$, $k_{26} = 2.29 \times 10^6$, $k_{56} = 4.43 \times 10^9$, $k_{57} = 1.08 \times 10^7$, $k_{47} = 2.60 \times 10^4$, $k_{78} = 3.98 \times 10^6$, $k_{86} = 3.40 \times 10^8$, $k_{79} = 6.83 \times 10^8$, $k_{510} = 9.76 \times 10^6$, $k_{611} = 9.76 \times 10^6$, $k_{911} = 3.82 \times 10^6$, $k_{1011} = 4.73 \times 10^9$, $k_{21} = 2.60 \times 10^4$, $k_{32} = 7.34 \times 10^6$, $k_{13} = 1.66 \times 10^{10}$, $k_{43} = 3.58 \times 10^3$, $k_{51} = 5.44 \times 10^5$, $k_{64} = 1.08 \times 10^7$, $k_{62} = 5.44 \times 10^5$, $k_{65} = 2.60 \times 10^4$, $k_{75} = 9.76 \times 10^6$, $k_{74} = 4.73 \times 10^9$, $k_{87} = 2.29 \times 10^6$, $k_{68} = 3.58 \times 10^3$, $k_{97} = 9.76 \times 10^6$, $k_{105} = 1.08 \times 10^7$, $k_{116} = 1.08 \times 10^7$, $k_{119} = 1.79 \times 10^3$, $k_{1110} = 2.60 \times 10^4$.

Three-dimensional profile

To show the overall trends of state transitions, a state-and-barrier profile was drawn. The rate constants at 150 mM [K^+] are converted using the following equation,

$$k^{\ddagger} = -\text{Log}\left[\frac{k}{\kappa\, k_B\, T/\hbar}\right]$$

where k is the rate constant, κ the transmission coefficient, k_B the Boltzmann constant, T the absolute temperature, and h, the Planck constant. This value is plotted on the vertical axis, and the transition paths are connected to form a profile (Figure S1). The zero level is defined arbitrary for the state 2 level. Transitions between states are shown with deflected lines. For example, the transition between state 10 and 11 is shown as a red path rising from state 10, reaching a peak and declining to state 11. The height from the level of state 10 to the peak represents the log[rate constant] of the transition from state 10 to 11 (k1). The asymmetric shape of the path indicates that the transition barrier from state 11 to 10 is higher than that of the reverse transition. At the bottom of the 3D profile, the model diagram is shown (orange footprint).

The state-and-barrier profile for the wild-type channel (Supplementary Fig. S1a) showed a tendency that K^+ binding to the wide-pore K^+ binding site (state 2, 4, 6 and 11) is more stable compared with other states. This finding indicates that the wide-pore K^+ binding site is mostly occupied at this concentration (150 mM [K^+]). The profiles of rate constants in the mutant (Supplementary Fig. S1b) were different from those of the wild-type channel. For example, the rate of binding to the wide-pore K^+ binding site (transfer from state1 to state 2, orange arrow, Supplementary Fig. S1) was increased in the mutant.

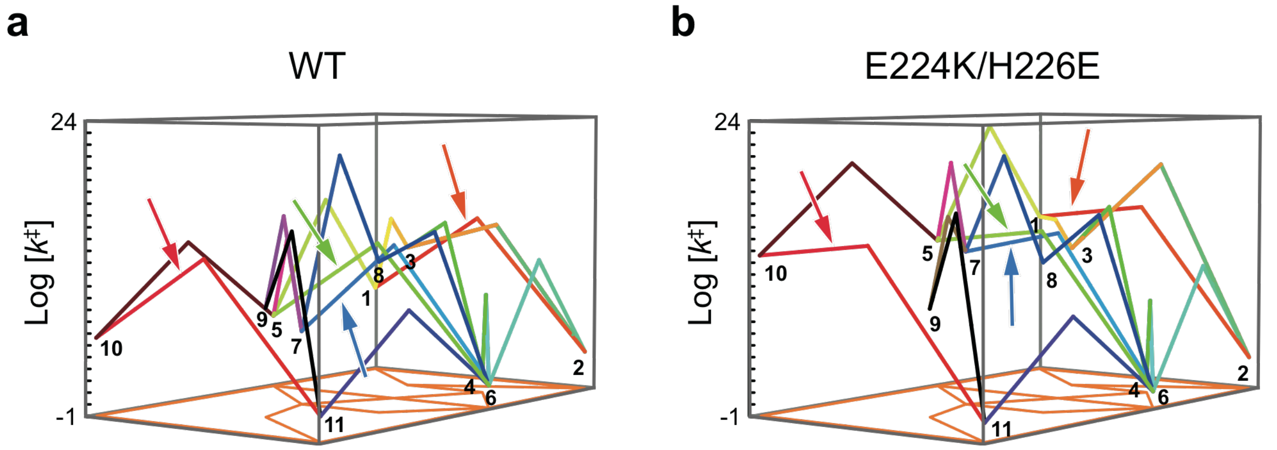

Supplementary Figure S1. Barrier-and-peak profile for the wild-type (**a**) and the mutant (**b**). The blue arrow highlights that transition rate from state 7 to state 4 was increased; green arrow points out that transition rate from state 5 to state 6 was increased; red arrow indicates that transitions from state 10 to state 11 was increased in the mutant.

Voltage-dependency of the relative cycle contributions

The relative cycle contributions of cycles at −100, 0, and +100 mV for the wild-type and mutant channels are shown in Supplementary Fig. S2. In the wild-type channel, cycle ***a*** was predominant at negative potentials, but gradually decreased its contribution as the membrane potential became more positive. Cycle ***c*** became predominant, and cycle ***d*** and ***e*** started to contribute slightly at more positive voltages. In the mutant channel, cycle ***a–c*** contributed throughout the voltage range tested. Cycle ***c*** having the $ccr_{\text{w-i}}$ value of 2 became predominant at positive potentials.

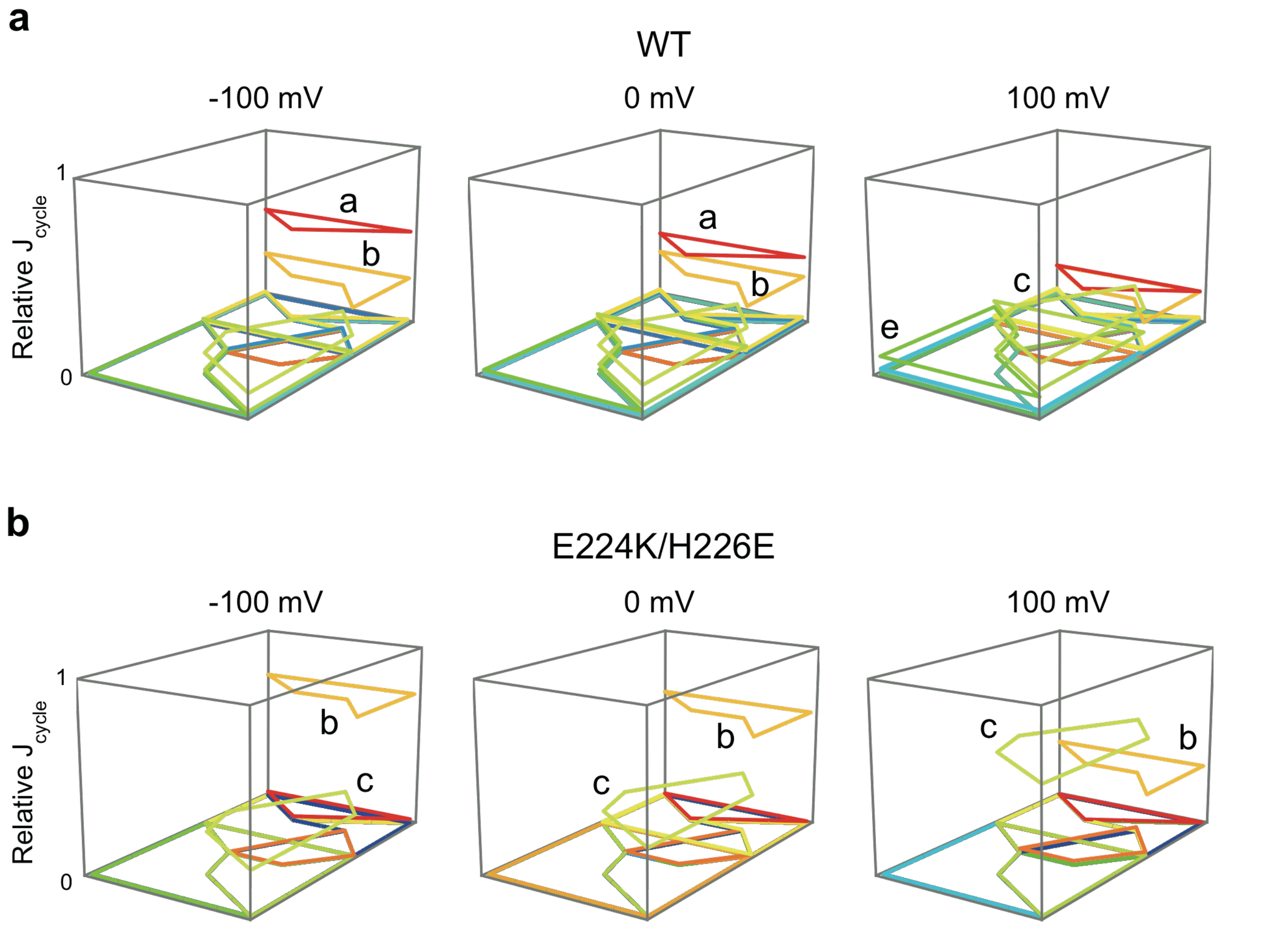

Supplementary Figure S2. Voltage dependency of the relative contribution of cycles. a. The relative cycle contribution for the wild-type channel at −100, 0, and +100 mV. **b.** The relative cycle contribution for the E224K/H226E mutant channel at −100, 0, and +100 mV. The concentration of K^+ is 150 mM.

Offline capacitance compensation

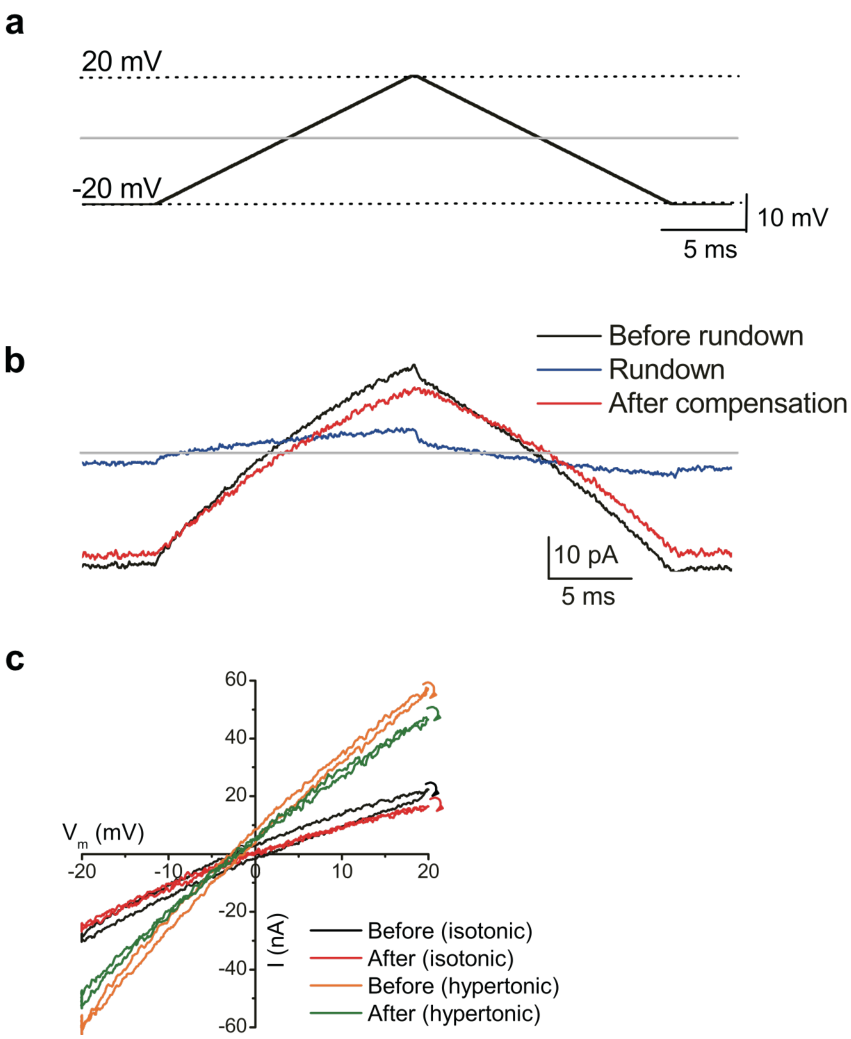

Supplementary Figure S3. Offline capacitance compensation. a. Voltage protocol. **b**. Currents recorded before (black) and after (blue) channel rundown. Currents after capacitance subtraction are shown in red. **c**. Currents before and after capacitance current subtraction in the absence (null △Osm) and presence of △Osm.

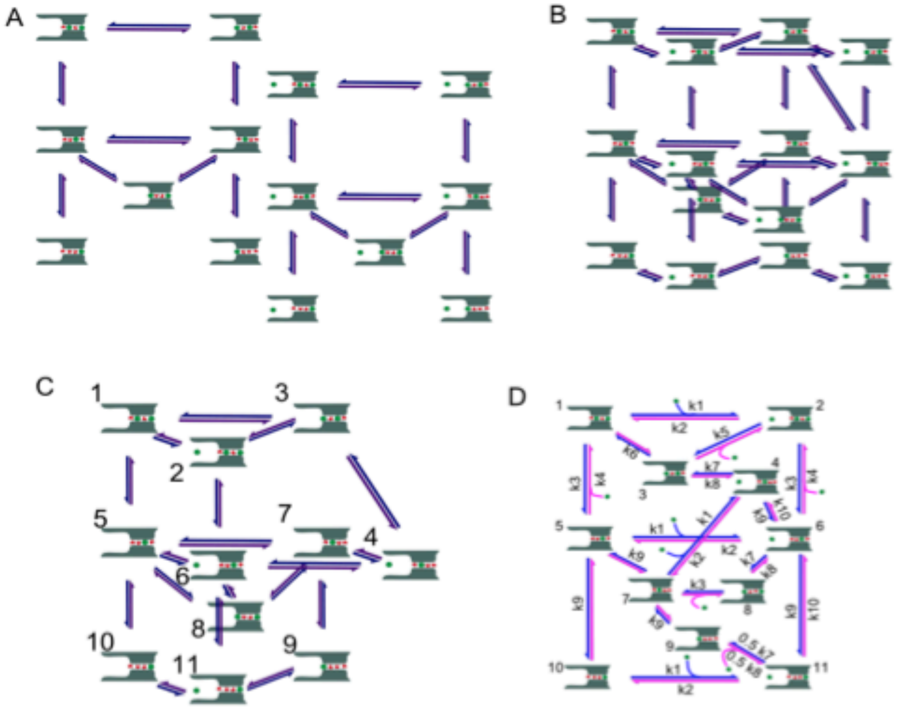

Supplementary Figure S4. The model building process from a simple canonical model. **a**. Two separate models (7-state models) with (right panel) and without (left panel) an ion in the wide pore. The transition paths between states 9 and 10 (left panel) and states 9’ and 11 (right panel) were deleted for physical consistency of the permeation process. **b**. Combining these models yielded a model involving ion permeation process in the wide pore, which is a kind of two-tier model, having a 3D architecture. **c**. To simplify the model, similar degenerative states and accompanying transition paths were deleted, which still kept three dimensionality. **d**. The stereoscopic expression of the states was projected in a fashion similar to the original degenerative way of the model proposed by Morais-Cabral et al.
